# P04 Dual pH/enzyme-responsive capsules of ciprofloxacin; a strategy to maximize the efficacy against infection causing superbugs such as MRSA and *Pseudomonas aeruginosa*

**DOI:** 10.1093/jacamr/dlac133.008

**Published:** 2023-01-19

**Authors:** Joshua C Nwabuife, Calvin A Omolo, Eman Ismail Abdallah, Mohammed Ali Gafar, Mahir Mohammed, Sanjeev Rambharose, Thirumala Govender

**Affiliations:** Discipline of Pharmaceutical Sciences, College of Health Sciences, University of KwaZulu-Natal, Durban 4000, South Africa; Discipline of Pharmaceutical Sciences, College of Health Sciences, University of KwaZulu-Natal, Durban 4000, South Africa; Department of Pharmaceutics, School of Pharmacy and Health Sciences, United States International University-Africa, P. O. Box 14634–00800, Nairobi, Kenya; Discipline of Pharmaceutical Sciences, College of Health Sciences, University of KwaZulu-Natal, Durban 4000, South Africa; Discipline of Pharmaceutical Sciences, College of Health Sciences, University of KwaZulu-Natal, Durban 4000, South Africa; Discipline of Pharmaceutical Sciences, College of Health Sciences, University of KwaZulu-Natal, Durban 4000, South Africa; Department of Physiological Sciences, Faculty of Science, Stellenbosch University, Private Bag X1 Matieland, Stellenbosch 7602, South Africa; Discipline of Pharmaceutical Sciences, College of Health Sciences, University of KwaZulu-Natal, Durban 4000, South Africa

## Abstract

**Background:**

Antimicrobial resistance is a public health problem of global concern and there is a high turnover of antibiotics developing resistance when compared with new drugs being developed. Therefore, there is a need to develop multifunctional systems that protect and potentiate the current drug on the market. Herein, we present novel dual pH/enzyme-responsive capsules for ciprofloxacin free base (CFB) delivery against MRSA and *P. aeruginosa*.

**Methods:**

Nano capsules preparation was carried out using the interfacial deposition method and was characterized for their physicochemical properties, as well as binding activities to HAase, and *in vitro* antibacterial properties. The preliminary biocompatibility was also evaluated via MTT assay.

**Results:**

The cytotoxicity results revealed the viability of the MCF-7 celines to be above 75% after exposure to the prepared capsules, therefore indicating its biosafety. Capsules had hydrodynamic diameter (*DH*), polydispersity index (PDI) and a surface-charge (ζ) of 261.4 ± 0.70 nm, 0.26 ± 0.01, and −15.2 ± 2.36 mV at pH 7.4 and 258.9 ± 2.51 nm, 0.28±0.01, and −5.27 ± 0.12 mV at pH 6 respectively, with an entrapment efficiency (EE%), of 85.63 ± 2.83%. Results from the release study indicated a faster CFB release from the prepared capsules at pH 6 compared with pH 7.4 confirming pH-responsive drug release. Also, upon the addition of HAase enzyme, the release rate of CFB at pH 7.4 was faster compared with the capsules without the enzyme at the same pH. MST results showed the prepared capsules to have a higher binding activity to bacterial enzyme HAase compared with the natural substrate with Kd of 0.1325 ± 0.0099 μM. Capsules were seen to show a better antibacterial efficacy compared with bare CFB with 16-fold and 128-fold recorded at pH 7.4; 128-fold and 8-fold recorded at pH 6, against MRSA and *P. aeruginosa,* respectively. Intracellular component leakage studies revealed the capsules to destroy the cell wall of the bacteria, thereby causing a greater reduction in the quantity of intracellular constituents such as protein and DNA, compared with bare CFB. Also, capsules showed better inhibition of efflux pump by a show of higher fluorescence intensity compared with bare CFB.

**Conclusions:**

Thus, the prepared capsules are a potential nano delivery system for CFB antibiotics, with the potential of improving the antibacterial efficacy of CFB against infections and disease condition induced by MRSA and *P. aeruginosa*.

